# Mapping Six New Susceptibility to Colon Cancer (*Scc*) Loci Using a Mouse Interspecific Backcross

**DOI:** 10.1534/g3.112.002253

**Published:** 2012-12-01

**Authors:** Chevonne D. Eversley, Xie Yuying, R. Scott Pearsall, David W. Threadgill

**Affiliations:** * Department of Genetics, Center for Gastrointestinal Biology and Disease, and Lineberger Cancer Center, University of North Carolina, Chapel Hill, North Carolina 27599; † Department of Genetics, North Carolina State University, Raleigh, North Carolina 27695

**Keywords:** spretus, cancer modifier, colorectal cancer

## Abstract

Colorectal cancer (CRC) has a complex etiology resulting from the combination of multiple genetic and environmental factors, each with small effects. Interactions among susceptibility modifier loci make many of the loci difficult to detect in human genome-wide association studies. Previous analyses in mice have used classical inbred strains, which share large portions of their genomes due to common ancestry. Herein, we used an interspecific backcross between the *Mus musculus* strain A/J and the *Mus spretus* strain SPRET/EiJ to map 6 additional CRC modifier loci (*Scc16-21*) and 2 suggestive loci. Three loci modify the location of tumors along the proximal-distal axis of the colon. Six CRC modifiers previously mapped in intraspecific crosses were also replicated. This work confirms genetic models suggesting that CRC is caused by many small effect alleles and brings the catalog of reported CRC modifier loci to 23 spread across 13 chromosomes. Furthermore, this work provides the foundation for large population-level epistatic interaction tests to identify combinations of low effect alleles that may have large effects on CRC susceptibility.

Colorectal cancer (CRC) is the third most commonly diagnosed form of cancer in developed countries, and it is responsible for the second largest number of cancer-related deaths. First-degree family members of an individual with sporadic cancer have a 2- to 3-fold increased risk of developing CRC (Bishop and Thomas 1990; Stephenson *et al.* 1991), indicating that shared genetic factors likely contribute to differential susceptibility. Twin studies demonstrate that heritable factors account for as much as 35% of sporadic CRC cases (Lichtenstein *et al.* 2000).

Genetic analyses in both humans and mice suggest that multiple small-effect alleles contribute to sporadic CRC susceptibility. Recent genome-wide association studies in humans have identified loci that increase risk associated with CRC (Broderick *et al.* 2007; Houlston *et al.* 2008; Jaeger *et al.* 2008; Tomlinson *et al.* 2007, 2008; Zanke *et al.* 2007). In an independent study, three of the loci were replicated, and combined analysis suggested that the odds ratio is at most 2.6 for a high-risk patient with two risk alleles among the three loci (Tenesa *et al.* 2008). Similar to the variability in susceptibility of humans to sporadic CRC, different mouse strains show varying susceptibility to colonic tumors (Bissahoyo *et al.* 2005; Diwan and Blackman 1980).

The CRC susceptibility loci identified in mice have primarily been based upon the azoxymethane (AOM) or related dimethylhydrozine (DMH) carcinogen model (Schauer *et al.* 1969; Uronis and Threadgill 2009; Ward 1975). AOM induces tumors in the distal mouse colon that resemble sporadic CRC of the descending colon in humans both histologically (Chang 1978; Uronis *et al.* 2007) and molecularly (Hirose *et al.* 2003; Kaiser *et al.* 2007; Takahashi *et al.* 2000; Vivona *et al.* 1993). Susceptibility to colon cancer 1 (*Scc1*) was one of the first low-penetrance cancer modifiers to be successfully mapped and cloned in mice using the AOM/DMH model (Ruivenkamp *et al.* 2002). An additional 14 *Scc* loci have been mapped using the same recombinant congenic panel between BALB/cHeA and STS/A strains (Moen *et al.* 1992, 1996; Ruivenkamp *et al.* 2003; van Wezel *et al.* 1999), and more recently, one unnamed locus using genome-wide association in a panel of inbred mouse strains (Liu *et al.* 2012). Two additional colon carcinogenesis susceptibility loci, *Ccs1* and *Ccs2*, were reported in studies using crosses between ICR/Ha and C57BL/6Ha and between CBA/J and C57BL/6J, respectively (Angel *et al.* 2000; Jacoby *et al.* 1994).

A recent analysis of inbred mouse strains revealed that common laboratory strains have a significant, shared contribution from *Mus musculus domesticus* (Roberts *et al.* 2007; Yang *et al.* 2011). The consequence of common ancestry is that large genomic intervals are identical by descent, resulting in reduced genetic diversity. Consequently, previous studies in mice have analyzed only part of the mouse genome for CRC susceptibility loci. SPRET/EiJ (*M. spretus)* diverged from *M. musculus* over one million years ago and offers the potential to analyze additional regions of the mouse genome. Using genetic crosses between the AOM-resistant SPRET/EiJ strain and the susceptible A/J strain, we mapped six additional *Scc* loci and two suggestive loci influencing a variety of tumor phenotypes, including position along the proximal-distal axis of the colon.

## MATERIAL AND METHODS

### Genetic crosses

A/J (A) and SPRET/EiJ (S) mice were obtained from the Jackson Laboratory (Bar Harbor, ME). Female A/J mice (*M. musculus)* were crossed to male SPRET/EiJ mice (*M. spretus)* to generate ASF1 hybrids. Since this interspecific cross generates infertile males, ASF1 females were backcrossed to A/J males to create a population of 235 (ASF1)A N2 mice.

### Cancer phenotyping

Mice, two-to-four months of age, were given four weekly intraperitoneal injections of AOM at 10 mg/kg of body weight (Sigma-Aldrich, St. Louis, MO) as previously determined to optimize differential susceptibility to AOM-induced CRC (Bissahoyo *et al.* 2005). Mice were weighed and killed by CO_2_ asphyxiation 20 weeks after the last AOM dose. A tail clip, liver lobe, and the entire colon were removed from each mouse. Colons were gently flushed with phosphate buffer saline, mounted on bibulous paper, and splayed open along the longitudinal axis. The number of tumors, their sizes, and locations along the proximal-distal axis were recorded.

### Genotyping

DNA was extracted from liver or tail samples from each mouse using Puregene DNA Purification Kit (Promega, Madison, WI). Mice from the (ASF1)A N2 generation were genotyped using a custom Sequenom MassARRAY SNP Genotyping platform containing 183 single nucleotide polymorphisms (SNP) markers (GeneSeek, Lincoln, NE). The custom platform design included SNP markers from NCBI Build 37 spaced at 10–15 cM intervals selected to be informative between the A/J and SPRET/EiJ mouse strains. All genotype and phenotype data are provided in Supporting Information, File S1.

### QTL mapping

Genotype probabilities were calculated using the Haldane map algorithm in J/qtl software (http://research.jax.org/faculty/churchill/software/Jqtl/index.html), a Java interface for R/qtl (Broman *et al.* 2003). As none of the tumor phenotypes could be transformed to fit a Gaussian distribution when all mice were included, the binary phenotype of presence or absence of tumors was used for the initial analysis. Binary analysis was performed by obtaining the maximum-likelihood estimates using the EM algorithm at 2 cM intervals throughout the genome (Broman 2003). Mice without tumors were excluded from further quantitative analyses. Genetic analysis of body weight was performed on nontransformed data using all mice.

The phenotypic measurements tumor number, average tumor diameter, maximum tumor diameter, and tumor position along the proximal-distal colonic axis from mice with tumors (n = 113) were transformed using rank z-transformation parameters and analyzed as normally distributed phenotypes. One-dimensional genomes scans were performed using the EM algorithm. Tumor load (tumor number × average tumor diameter) could not be transformed to fit a normal distribution and was analyzed using an extension of the Wilcoxon rank sum test for non-parametric interval mapping (Broman 2003; Kruglyak and Lander 1995). No secondary loci were detected in any of the one-dimensional genome scans when controlling for the primary locus that was detected in each analysis. Only loci that reached genome-wide significance were given official designations.

Two-dimensional analysis of tumor phenotypes was performed using Haley-Knott regression algorithms to identify epistatic interactions. Significance thresholds were calculated by performing 1000 permutations with intervals set at the 182 SNP markers used in the analysis. The additive and interaction models were used to determine the type of epistasis influencing phenotypic variance.

Pointwise Wilcoxon rank sum test was performed on SNP markers in close proximity to previously identified *Scc* loci (Moen *et al.* 1992, 1996; Ruivenkamp *et al.* 2003; van Wezel *et al.* 1999).

## RESULTS

### Validation of the A/J × SPRET/EiJ cross for modifier mapping

**Figure 1:**
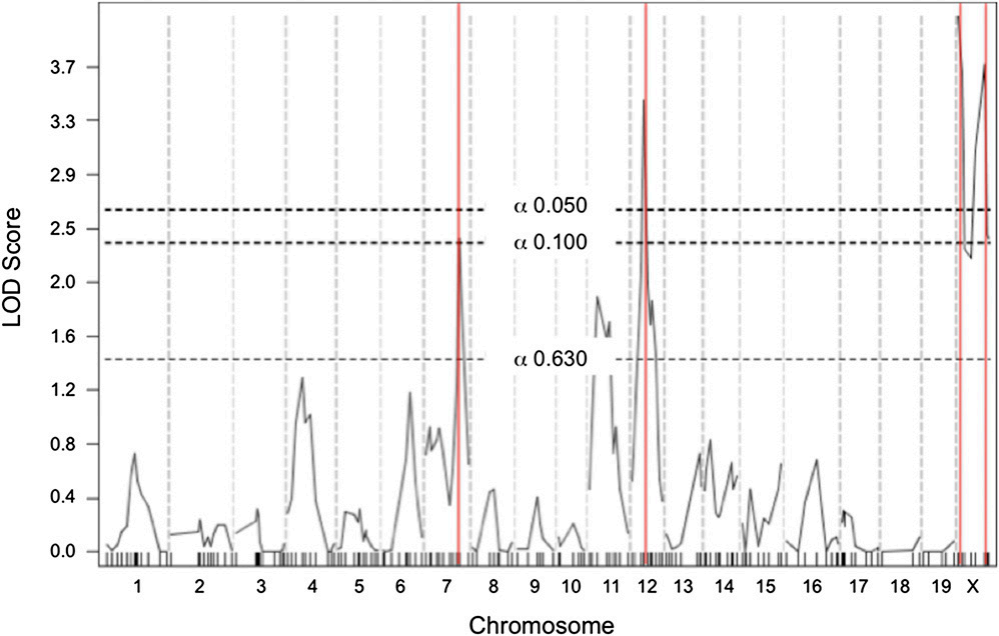
Genome scan for modifier loci controlling body weight. Significance thresholds are shown by dotted lines, and red lines mark peak LOD scores.

To validate the utility of the interspecific backcross population for mapping modifiers of CRC susceptibility, modifier loci controlling body weights of (ASF1)A N2 mice were analyzed, and their locations were compared with previously identified obesity modifiers in SPRET/EiJ crosses. Loci on Chr 7 (*Mob1*) and Chr12 (*Mob3*), originally reported in a (C57BL/6J × SPRET/EiJ) × C57BL/6J backcross (Fisler *et al.* 1993; Warden *et al.* 1995; Yi *et al.* 2004), were replicated (Figure 1). Additionally, two previously reported body weight modifiers on the X chromosome (*Bw1* and *Bw3*) were replicated. *Bw1* and *Bw3* were previously reported in (A/J × SPRET/EiJ) × C57BL/6J and (C3H/He × SPRET/EiJ) × C57BL/6J crosses, respectively (Dragani *et al.* 1995).

### Replication of previously mapped *Scc* loci

**Figure 2:**
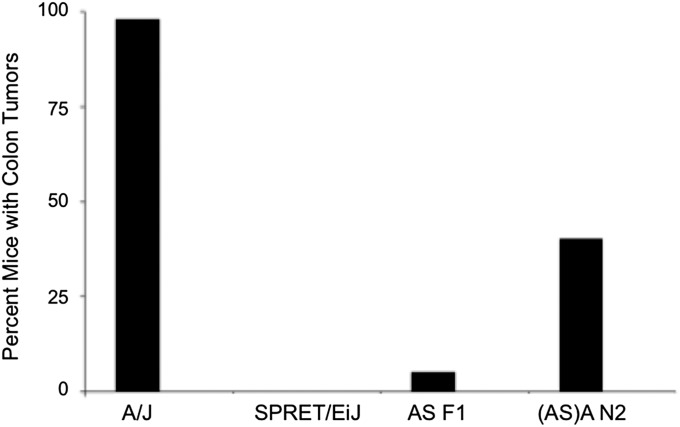
Colon tumor penetrance of parental strains, F1 hybrids, and N2 backcross mice used in the current study.

When exposed to four weekly doses of AOM, 100% of A/J mice developed colonic tumors, whereas SPRET/EiJ mice were completely resistant (Figure 2). Genome-wide, SPRET/EiJ is dominant to the susceptible A/J strain, as less than 5% of ASF1 hybrids develop tumors. Almost 50% of the (ASF1)A N2 backcross mice (113 of 235) developed colorectal tumors in response to AOM exposure.

To determine whether previously mapped colon tumor susceptibility loci also contribute to differential susceptibility in the (ASF1)A N2 population, point-wise analysis was performed using the SNP marker nearest to previously identified *Scc* and *Ccs* loci (Table 1) (Angel *et al.* 2000; Jacoby *et al.* 1994; Moen *et al.* 1992, 1996; Ruivenkamp *et al.* 2003; van Wezel *et al.* 1996, 1999). Markers near *Scc9*, *Scc11*, *Scc13*, and *Scc15* showed significant association with tumor presence, whereas *Scc5* and *Scc13* were associated with tumor number and tumor load. *Scc8* was only associated with tumor load. No other *Scc* or *Ccs* loci showed an association with colon tumor phenotypes in the (ASF1)A N2 population, and none of the previously mapped loci reached significance when performing a genome-wide analysis, consistent with being low-effect susceptibility alleles. Since SNPs were not selected based on location of previous colon cancer susceptibility loci, some markers were more distant, resulting in reduced power to replicate previous results.

**Table 1:** Point-wise analysis of previously mapped colon cancer susceptibility loci

Locus	SNP Marker	Chr	Mb	Presence/Absence *P*-value	Tumor Multiplicity *P*-value	Tumor Load *P*-value
*Scc1*	rs3716390	2	81.6	0.55	0.81	0.41
*Scc2*	rs4223152	2	52.5	0.64	0.45	0.72
*Scc3*	rs8253293	1	196.9	0.28	0.37	0.13
*Scc4*	rs4231637	17	73.5	0.13	0.93	0.78
*Scc5*	UNC_18_37970902	18	37.9	0.66	**0.02**	**0.02**
*Scc6*	rs4228590	11	17.1	0.74	0.11	0.15
*Scc7* / *Ccs2*	rs6322812	3	159.5	0.08	0.45	0.73
*Scc8*	rs3701395	8	3.1	0.25	0.09	**0.02**
*Scc9*	rs13480754	10	106.9	**0.05**	0.47	0.70
*Scc10*	rs16822005	2	91	0.42	0.85	0.41
*Scc11*	rs3024208	4	130.4	**0.03**	0.09	0.25
*Scc12*	rs16807506	7	135	0.53	0.69	0.85
*Scc13*	rs29973570	6	72.5	**0.03**	**0.03**	**0.02**
*Scc14*	rs6280091	10	31.4	0.54	0.66	0.87
*Scc15*	rs4228762	11	58	**<0.01**	0.11	0.18
*Ccs1*	rs8261201	12	88.5	0.24	0.12	0.35

*P*-values lower than 0.05 are marked in bold.

### SPRET/EiJ harbors a major colon cancer resistance locus

**Figure 3:**
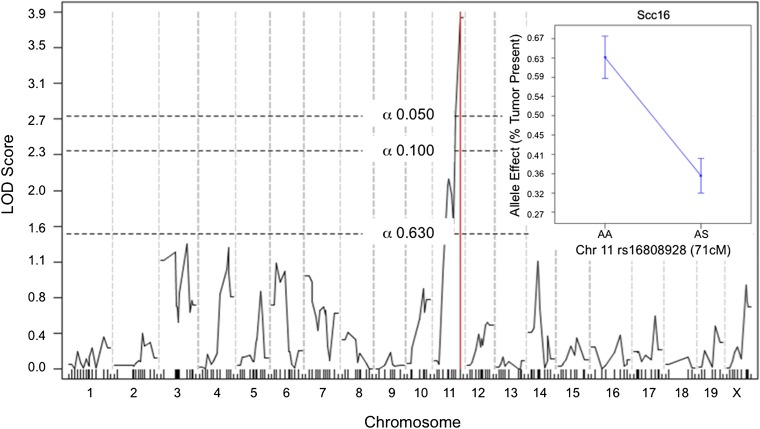
Genome scan for modifier loci controlling the binary tumor presence phenotype. Significance thresholds are shown by dotted lines, and red line marks peak LOD score. Inset shows allele effect plot of the peak marker on Chr 11.

Binary analysis of tumor presence (≥1 tumor, n = 113 mice) and absence (0 tumors, n = 125 mice) detected a single modifier locus (LOD = 3.83; Figure 3). This locus, designated *Scc16* (Table 2), was most strongly associated with rs16808928 (Chr 11, 71 cM). A tumor effect plot revealed that SPRET/EiJ contributes a tumor-resistance allele at *Scc16* (Figure 3, inset); mice heterozygous at rs16808928 have a significantly increased likelihood of being tumor free than mice homozygous for the A/J allele (*t* test, *P* < 0.0001).

**Table 2:** Summary of colon cancer modifier loci identified in an (ASF1)A N2 backcross

Locus	Phenotype	SNP Marker	Chr	Mb*^a^*	Conf Int (cM)*^b^*	LOD Score	*P*-value
*Scc16*	Tumor presence	rs16808928	11	116.4	32.0–71.0	3.83	<0.01
*Scc17*	Tumor number	rs13479769	8	55.6	12.0–70.8	2.66	0.04
Chr 1	Tumor number	rs3658044	1	19.2	0–20.0	2.291	0.11
*Scc18*	Tumor load	rs16805672	6	88.7	36.2–58.7	2.825	0.04
*Scc19*	Max tumor size × sex/tumor load	rs13482118	14	30.6	5.8–20.2	3.389	0.05
*Scc20*	Rel distal position × tumor number	rs8238935	1	58.1	9.1–34.5	4.067	<0.01
						Full / Additive	Alpha
*Scc17* × *Scc21*	Tumor load	rs13479769 × rs16810780	8 × 1	55.6 × 34.5		5.565 / 4.581	0.10
*Scc18* × *Scc19*	Tumor load × sex	rs16805672 × rs13482118	6 × 14	88.7 × 30.6		6.236 / 6.190	0.05
						Full / Interaction	Alpha
Chr 1 × Chr 10	Rel distal position × tumor number	rs3658044 × NCBI_10_99187828	1 × 10	19.2 × 99.1		7.358 / 6.351	0.10

a Physical location of SNP markers.

b 1.5 LOD confidence intervals based on genetic analysis.

### Colorectal cancer phenotypes are determined by oligogenic modifier loci

Mice without tumors were excluded from analysis of quantitative tumor phenotypes, including tumor number, tumor load, average tumor diameter, maximum tumor diameter, and tumor position along the colon, phenotypes that are weakly correlated within the (ASF1)A N2 backcross population (Table 3). Only average tumor diameter and maximum tumor diameter, as well as tumor number and tumor load have *r^2^* values greater than 0.7. The fact that average tumor size was not correlated with tumor load, which is the product of average tumor size and tumor number, suggests that tumor number is much more variable than average tumor diameter and is the major determinant of tumor load.

**Table 3:** Tumor phenotype correlation matrix

*r*^2^	Tumor Load	Max Tumor Diameter	Avg Tumor Diameter	Relative Position	Tumor Number
Tumor load	1.00	0.68	0.50	0.47	0.89
Max tumor diameter		1.00	0.86	0.31	0.47
Avg tumor diameter			1.00	0.17	0.23
Relative position				1.00	0.45
Tumor number					1.00

Tumor number is the only phenotype that was not transformable into a normal distribution. Non-parametric methods detected a single modifier on Chr 8 named *Scc17* (rs13479769, 30 cM; LOD = 2.66; Table 2), which explains 10.1% of the variance in tumor number (*P* = 0.001). An effect plot revealed that mice heterozygous at the *Scc17* locus have fewer tumors than mice homozygous for the A/J allele (*t* test, *P* = 0.0003), showing that the A/J allele increases tumor number. An additional putative tumor number modifier on Chr 1 (rs3658044) was suggestive; this locus potentially explains 7.6% of the phenotypic variance (*P* = 0.003).

Analysis of tumor load also identified one modifier on Chr 6 named *Scc18* (rs16805672, 37 cM; LOD = 2.826; Table 2), which explains 11.4% of the variance (*P* < 0.0001). Interestingly, the SPRET/EiJ *Scc18* allele increases tumor load. No loci were detected that modified average or maximum tumor diameter. However, a sex-dependent modifier locus on Chr 14 named *Scc19* was detected for maximum tumor size (LOD = 3.421); *Scc19* explains less than 1% of the variance (*P* = 0.325).

**Figure 4:**
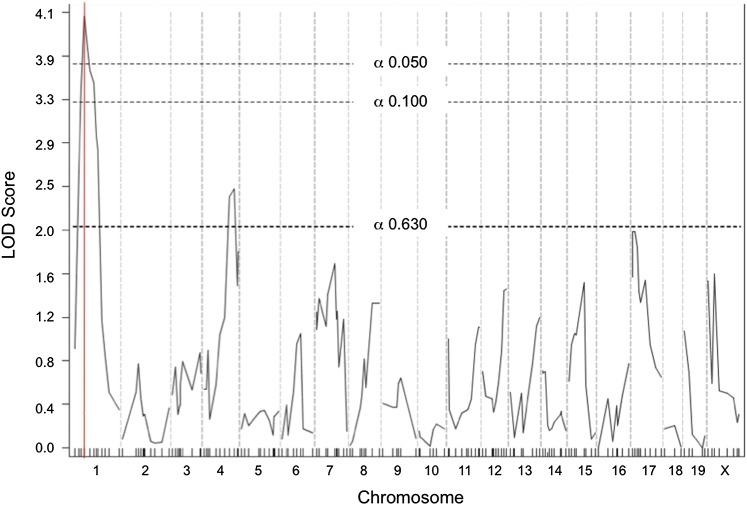
Genome scan for modifier loci controlling the tumor number phenotype. Significance thresholds are shown by dotted lines, and red line marks peak LOD score.

The colon has a proximal-distal axis with regional differences in cell composition and function. Modifiers influencing tumor position within the colon have not been reported. To identify whether modifiers influence tumor position, a genome scan was performed using measurements of tumor position along the proximal-distal axis of the colon. Although no significant associations were detected using tumor position alone, if tumor number were considered as a covariate, one modifier named *Scc20* was identified on Chr 1 (rs8238935, 32 cM; LOD = 4.067; Figure 4). This locus is proximal to the suggestive tumor number modifier Chr 1 (rs3658044) with an overlapping confidence interval; however, it is likely to be a distinct locus because the *r^2^* correlation between tumor number and position is only 0.45 (Table 3). Mice homozygous for the A/J *Scc20* allele are likely to have tumors that are positioned more distally than mice heterozygous at the *Scc20* locus.

### Epistatic interactions among modifiers

**Figure 5:**
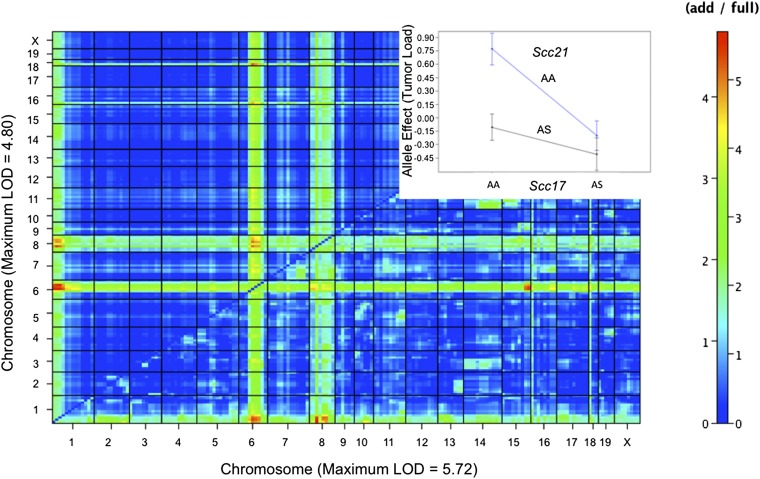
Heat plot of two-dimensional genome scan for tumor load modifiers. Color scale represents significance levels. The image above the diagonal is the additive portion of allele effect and below the diagonal is the full allele effect. Inset shows effect plot of the interaction detected between Chr 8 and 1 (*Scc17* × *Scc21*).

Two-dimensional modifier scans were performed on all tumor phenotypes to detect allele combinations that may have gone undetected using one-dimensional scans. Analysis of tumor load revealed one significant (α = 0.05) and one suggestive interaction (α = 0.1; Figure 5 and Table 2). The suggestive additive interaction was detected between Chr 8 (*Scc17*) and a locus on Chr 1 named *Scc21* (full LOD = 5.565, and additive LOD = 4.581). An effect plot of tumor load shows that the tumor load–increasing effect of the A/J allele at *Scc21* is observed only when mice are also homozygous for the A/J allele at *Scc17* (Figure 5, inset). A larger study will be required to validate putative epistatic interactions between loci on Chrs 1 and 6, Chrs 6 and 8, and Chrs 6 and 18 that do not reach the suggestive threshold set for interactions (Figure 5), as well as for the detection of interactions previously reported such as between *Ssc7* and *Scc8* (van Wezel *et al.* 1996).

When sex was used as a covariate with tumor load, a significant interaction was detected between *Scc18* and *Scc19* (full LOD = 6.236, and additive LOD = 6.190, Table 2).

A suggestive interaction was also detected for tumor position, but only if tumor number was used as a covariate (Table 2). The interaction LOD between (rs3658044) and a locus on Chr 10 (NCBI_10_99187828) is 7.358 (full–add).

## DISCUSSION

**Figure 6:**
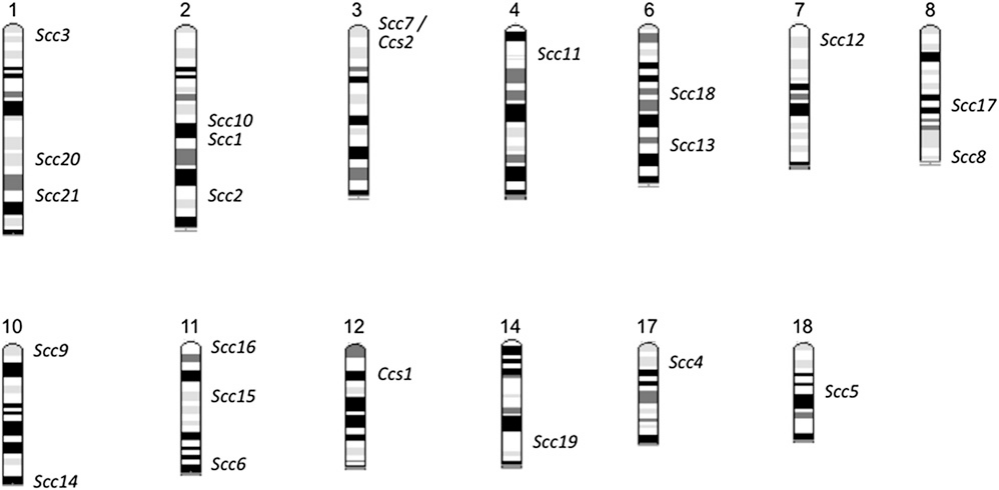
Comparative locations of all reported colon cancer susceptibility loci. Each locus is centered in its confidence interval.

Several loci influencing CRC susceptibility have been identified in crosses between resistant and susceptible mouse strains. Numerous studies using recombinant congenic strains led to the identification of 15 *Scc* loci, with almost half being involved in two-way interactions (Moen *et al.* 1992, 1996; Ruivenkamp *et al.* 2003; van Wezel *et al.* 1996, 1999). Analyses of additional crosses were used to identify *Ccs1* and *Ccs2* (Angel *et al.* 2000; Jacoby *et al.* 1994). We employed an interspecific backcross to replicate six of the previously reported loci and to identify six additional loci, *Scc16–21* (Figure 6). This brings the number of reported colon cancer susceptibility loci in mice to 23 spread across 13 chromosomes, with half of the known loci being detected in the interspecific backcross reported here. Similar to previous observations, numerous interactions between modifiers were observed, suggesting that networks of interacting, low-penetrance alleles determine CRC susceptibility. The small effect of most modifiers highlights the difficulties involved in identifying allelic combinations that influence cancer susceptibility in humans and that likely contributed to several previous modifier loci not replicating in the current study. Lack of replication could also be due to genetic differences among the strain combinations used and/or Type I errors.

A recently reported genome-wide association study using an intrasubspecific panel of mouse strains also replicated some previously detected *Scc* and *Ccs* loci (Liu *et al.* 2012), although only one, *Scc8*, was in common with the present study. The lack of concordance across the three approaches (intrasubspecific linkage, intrasubspecific association, and intersubspecific linkage) underscores the difficulties in detecting low-penetrance allele effects and the variation in loci detected that is dependent upon the genetic structure of the target populations.

Genome-wide analysis of an interspecific backcross between SPRET/EiJ and A/J reported here detected a locus (*Scc16*) with a major effect controlling penetrance of AOM-induced tumors. Tumor load was most strongly influenced by *Scc18* located on Chr 6. Furthermore, *Scc17*, modulating tumor number, and *Scc21* were found to interact to affect tumor load. Tumor load is also regulated in a sex-dependent fashion by *Scc19*, which was detected individually using maximum tumor diameter.

Modifiers also appear to influence the relative position of tumors along the proximal-distal axis of the colon. *Scc20* on Chr 1 and an interaction between suggestive loci on Chr 1 (rs3658044) and Chr 10 (NCBI_10_99187828) modify spatial positioning of tumors. Chr 10 (NCBI_10_99187828) is proximal to *Scc9* with both loci being located in a region of conserved synteny with human Chr 12. Interestingly, several studies report hypermethylation, overexpression, and chromosomal loss of genes associated with colon cancer on 12q (Nakao *et al.* 1998; Richter *et al.* 2003; van Dieren *et al.* 2006). The detection of Chr 10 (NCBI_10_99187828) in the present study increases the possibility that one or both of these loci represent alleles whose orthologs may be associated with cancer susceptibility in humans.

A number of genome-wide association studies have been conducted recently in humans, which revealed several common variants that function as low-penetrance susceptibility alleles. Although none of the mouse susceptibility loci identified here appear to be in conserved syntenic segments with those detected in the human studies, the mouse loci may function in similar pathways or may be detectable only when allele interactions are tested. Future identification of the underlying genes responsible for the *Scc* loci should reveal the relationship between mouse and human cancer susceptibility and how genetic modifiers influence susceptibility.

## Supplementary Material

Supporting Information
