# Supplementary material for: Mapping Six New Susceptibility to Colon Cancer (Scc) Loci Using a Mouse Interspecific Backcross
Source: G3 (Bethesda). 2012 Dec 1;2(12):1577–84. doi: 10.1534/g3.112.002253 (PMC3516479; doi:10.1534/g3.112.002253)
Supplement: Supporting Information [file supp_2_12_1577__index.html]

Supporting Information 

# Mapping Six New Susceptibility to Colon Cancer (*Scc*) Loci Using a Mouse Interspecific Backcross

## Supporting Information for Eversley *et al.*, 2012

**Files in this Data Supplement:**

- File S1 - Genotype and phenotype data (.xlsx, 179 KB)
